# Anesthesia Mumps With Transient Airway Compromise Following Spinal Anesthesia for Emergency Cesarean Delivery

**DOI:** 10.14740/jmc5347

**Published:** 2026-07-01

**Authors:** Egzon Daku, Rajmonda Nallbani, Fatos Sada, Leon B. Hajdari

**Affiliations:** a Faculty of Medicine, University of Prishtina, Prishtina, Kosova; b Department of Anesthesiology, Faculty of Medicine, University of Prishtina, Prishtina, Kosova; c Department of Anesthesiology, University Clinical Centre of Kosova, Prishtina, Kosova; d These authors contributed equally to this work.

**Keywords:** Anesthesia mumps, Spinal anesthesia, Cesarean section, Twin pregnancy, Parotitis

## Abstract

The authors present a 44-year-old woman who developed bilateral parotid swelling following spinal anesthesia for an emergency cesarean section. Shortly after surgery, the patient developed acute facial swelling associated with pain, trismus, and transient respiratory distress. Ultrasonographic evaluation was consistent with acute parotitis. Conservative management, including intravenous hydration, corticosteroids, analgesics, and supportive care, resulted in complete clinical recovery within 3 days. Although perioperative parotid swelling is an uncommon complication after spinal anesthesia, this case highlights its potential occurrence in obstetric patients and the possibility of transient airway compromise. Early recognition and prompt supportive management are essential to prevent complications and ensure favorable outcomes.

## Introduction

Acute parotid swelling and inflammation are an uncommon postoperative complication most frequently associated with general anesthesia and are commonly referred to in the literature as “anesthesia mumps.” Proposed mechanisms include dehydration, salivary duct obstruction, reduced salivary flow, and perioperative glandular stasis, which may predispose to transient parotid enlargement and inflammation [1, 2]. The occurrence of postoperative parotid swelling after spinal anesthesia is exceedingly rare, likely due to the absence of airway manipulation and reduced anesthetic exposure [3, 4]. Although usually self-limiting, rare cases of airway compromise requiring urgent intervention have been reported [5, 6].

We report a case of acute bilateral parotid swelling developing after emergency cesarean section performed under spinal anesthesia in a 44-year-old woman with twin pregnancy, highlighting the importance of early recognition and supportive management of this rare perioperative complication.

## Case Report

A 44-year-old woman, gravida 2 para 1 (G2P1), presented at 33 weeks and 5 days of gestation with a twin pregnancy. Her medical history was notable for a previous cesarean section, treatment for infertility, and one failed *in vitro* fertilization attempt. She had no known history of allergic reactions. One week prior to the emergency cesarean delivery, the patient was admitted with abdominal pain. She was managed with tocolytics and discharged after symptom resolution. She subsequently returned with infrequent uterine contractions, pelvic pain, and 1 cm cervical dilation. After 1 day of hospitalization, labor was induced. Due to breech presentation of one twin and ongoing pain, an emergency cesarean section was indicated. Preoperatively, the patient’s vital signs were stable, with a blood pressure of 138/74 mm Hg, heart rate of 112 beats per minute, oxygen saturation of 98% on room air, and body temperature of 36.4 °C. The cesarean section was performed under spinal anesthesia using a 25-gauge pencil-point needle following standard sterile precautions. Intrathecal administration at the L3–L4 interspace included 14 mg of 0.5% hyperbaric bupivacaine and 15 µg of fentanyl. The procedure was uncomplicated, lasting approximately 45 min, with an estimated blood loss of 500 mL. Intraoperatively, the patient received 1,500 mL of normal saline, 20 mg of ephedrine for the management of spinal anesthesia–induced hypotension, and 20 IU of oxytocin as a uterotonic agent.

**Figure 1:**
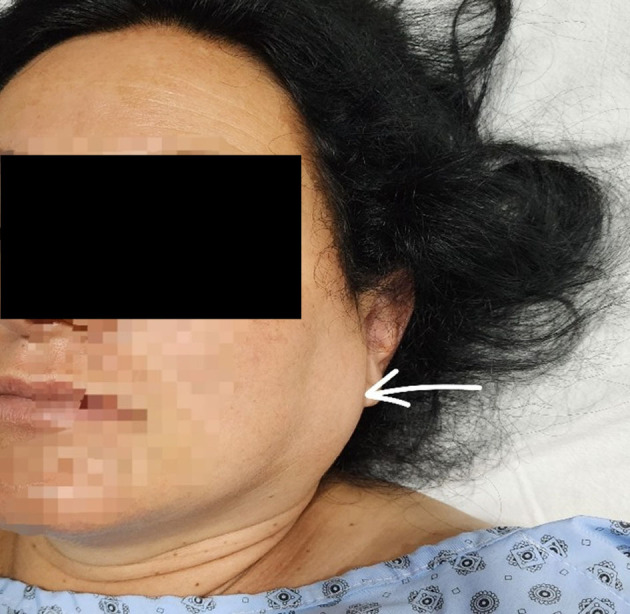
Left parotid region 2 h postoperatively, demonstrating unilateral gland enlargement with associated cervical soft-tissue edema (arrow).

Postoperatively, she was transferred to the obstetric intensive care unit for monitoring due to her high-risk obstetric status, including twin pregnancy and emergency cesarean delivery. Vital signs showed a blood pressure of 110/62 mm Hg, heart rate of 92 beats per minute, oxygen saturation of 99%, and body temperature of 36.1 °C. Postoperative management included intravenous fluid therapy, antibiotic administration, low-molecular-weight heparin, proton pump inhibitor therapy, and adequate analgesia. Approximately 2 h after surgery, the patient developed sudden-onset bilateral facial swelling and pain, predominantly involving the parotid gland regions (Fig. 1). Physical examination revealed bilateral parotid gland enlargement with tenderness on palpation, accompanied by edema extending to the temporal regions. The swelling had a fulminant onset and rapidly progressed, leading to difficulty in mouth opening and respiratory distress. Oxygen saturation levels subsequently decreased, necessitating assisted ventilation using a bag–valve–mask connected to supplemental oxygen. Throughout the episode, the patient remained conscious. She was afebrile, with no clinical evidence of trismus, neurological deficits, or allergic reaction (Fig. 1). During the acute phase, an allergic reaction was initially suspected. The patient was treated with intravenous methylprednisolone (40 mg), chlorpheniramine (10 mg), and dexamethasone (4 mg), while respiratory support was maintained using a bag–valve–mask with supplemental oxygen. The episode lasted approximately 30 min, after which the swelling gradually subsided and the patient’s respiratory symptoms improved. Following clinical stabilization, laboratory and imaging investigations were performed. Laboratory investigations conducted preoperatively and on the first postoperative day are summarized in Table 1. Preoperative values were within normal limits. On the first postoperative day, coinciding with the onset of facial swelling, there was mild leukocytosis with neutrophilic predominance and a marked elevation of C-reactive protein, consistent with an acute inflammatory response. An elevated neutrophil-to-lymphocyte ratio was also observed (Table 1). Ultrasonography of the parotid glands confirmed the diagnosis of acute parotitis, demonstrating diffuse glandular enlargement with hypoechoic areas consistent with inflammatory changes. Based on clinical and radiological findings, conservative management was continued, including adequate intravenous hydration and empiric antibiotic therapy with cefazolin and gentamicin. Analgesic therapy consisted of tramadol (100 mg) combined with metoclopramide (10 mg) and paracetamol (1 g). The patient was advised to maintain good oral hygiene and to perform regular massage of the affected parotid glands. The patient showed gradual clinical improvement over the following days, with complete resolution of parotid swelling and pain. Respiratory symptoms did not recur, and no adverse or unanticipated events were observed. The patient tolerated the treatment well, with good adherence to supportive measures. She was discharged in good general condition 3 days after surgery, with complete recovery of parotid gland function. No complications were noted during the hospital stay.

**Table 1:** Laboratory Values Preoperatively and on the First Postoperative Day

Parameter	Unit	Reference values	Preoperative	Postoperative day 1
White blood cell	× 10^3^/µL	4.0–10.0	9.5	13.4
Hemoglobin	g/dL	12.0–16.0	11.1	10.1
Hematocrit	%	36–46	38.0	31.5
Platelets	× 10^3^/µL	150–400	333	228
Monocytes	%	2–8	0.5	0.3
Granulocytes	%	40–70	7.0	12.2
Lymphocytes	%	20–50	2.0	0.9
C-reactive protein	mg/L	< 5	–	67.2

## Discussion

We describe a case of acute postoperative parotitis occurring after spinal anesthesia, a complication most commonly reported following general anesthesia and typically associated with airway manipulation, prolonged intubation, perioperative dehydration, and reduced oral intake [1, 7]. However, our case demonstrates that this rare condition can also develop in the absence of direct airway instrumentation. Comparable findings have been reported by Gautham et al, describing acute parotitis following neuraxial anesthesia [8], while Hamaguchi et al documented postoperative sialadenitis complicated by airway compromise after general anesthesia, underscoring the multifactorial nature of this condition [2].

In obstetric patients, fluid shifts, hormonal influences, and altered immune responses may further predispose to glandular inflammation. Tomlison et al emphasized the importance of careful perioperative fluid management, noting that inadequate hydration may contribute to salivary stasis and subsequent inflammation [9]. In our patient, despite spinal anesthesia, the combination of perioperative stress, relative dehydration, and hemodynamic fluctuations requiring vasopressor support likely contributed to reduced salivary flow and ductal stasis, facilitating the development of parotid swelling. Although anesthesia mumps is generally considered self-limiting, clinically significant airway compromise has been reported. Gunaydin et al described postoperative parotitis following cesarean delivery under spinal anesthesia [4], while Jarrar et al reported a similar presentation with transient respiratory distress managed conservatively [5]. In contrast, cases following general anesthesia described by Asghar et al and Udayakumar et al have demonstrated more severe courses requiring aggressive airway management, highlighting the variability in clinical severity [3, 6]. These findings suggest that the severity of anesthesia mumps is not solely dependent on the type of anesthesia, but rather on a combination of perioperative and patient-related factors. The pathophysiology of postoperative parotitis in the context of spinal anesthesia remains incompletely understood. Proposed mechanisms include sympathetic stimulation leading to salivary stasis, perioperative fluid imbalance, ductal obstruction, and transient ischemia [10–12]. Additional factors such as retrograde air entry into the salivary ducts (pneumo-parotitis), venous congestion in the cervicofacial region, and the effects of perioperative medications may further contribute to glandular swelling [13, 14]. In the present case, the rapid onset of symptoms within 2 h and the associated transient airway compromise suggests a multifactorial process, in which perioperative physiological stressors and altered salivary dynamics likely acted synergistically despite the absence of airway manipulation.

Management of anesthesia mumps is typically conservative, including adequate hydration, analgesia, anti-inflammatory therapy, and close airway monitoring [12]. In our patient, however, the condition was complicated by transient respiratory compromise, necessitating immediate assisted ventilation with a bag–valve–mask, although escalation to re-intubation or tracheostomy was avoided. Similar favorable outcomes with conservative management have been reported in the literature [11, 13–15]. Nevertheless, more severe presentations involving both parotid and submandibular glands have been described, resulting in massive cervicofacial edema and life-threatening airway obstruction requiring emergency airway intervention [16]. Importantly, this case demonstrates that anesthesia mumps, although often considered benign and self-limiting, can progress rapidly to clinically significant airway compromise requiring immediate ventilatory support.

This report adds to the limited literature on anesthesia mumps following spinal anesthesia in obstetric patients and highlights that the absence of general anesthesia does not eliminate the risk of airway involvement. Clinicians should maintain a high index of suspicion for sudden cervicofacial swelling and be prepared for prompt airway support and conservative management to prevent clinical deterioration

### Learning points

“Anesthesia mumps” is a rare postoperative complication that may occur even after spinal anesthesia and should be considered in patients presenting with acute postoperative cervicofacial swelling. Rapid bilateral parotid enlargement may lead to transient airway compromise, requiring immediate airway assessment and supportive management, including assisted ventilation when necessary. Early differentiation from allergic reactions, angioedema, and infectious parotitis is essential for appropriate treatment. Conservative management with adequate hydration, analgesia, corticosteroids, and supportive care is usually effective and associated with complete recovery. This case highlights the importance of maintaining a high index of suspicion and ensuring early recognition and prompt management to prevent potentially life-threatening complications and achieve favorable outcomes.

## Data Availability

The data supporting the findings of this study are available from the corresponding author upon reasonable request.

## AI Usage

The authors used an AI-based language model (ChatGPT, OpenAI) exclusively for language editing and clarity improvement. No AI tools were used for data analysis, interpretation, or generation of scientific content. All content was critically reviewed, validated, and approved by the authors.
